# Multicolor flow cytometric assessment of Ki67 expression and its diagnostic value in mature B-cell neoplasms

**DOI:** 10.3389/fonc.2023.1108837

**Published:** 2023-02-17

**Authors:** Xia Mao, Yi Li, Songya Liu, Cheng He, Shujuan Yi, Dong Kuang, Min Xiao, Li Zhu, Chunyan Wang

**Affiliations:** ^1^ Department of Hematology, Tongji Hospital, Tongji Medical College, Huazhong University of Science and Technology, Wuhan, China; ^2^ Department of Hematology, Renmin Hospital of Wuhan University, Wuhan, China; ^3^ Institute of Pathology, Tongji Hospital, Tongji Medical College, Huazhong University of Science and Technology, Wuhan, China

**Keywords:** multiparameter flow cytometry, Ki67, mature B-cell lymphoma, agreement, immunohistochemistry

## Abstract

**Background:**

There is no unified standard data about the sensitivity and specificity regarding flow cytometry analysis of Ki67 expression during lymphoma diagnoses.

**Objective:**

This evaluated the efficacy of multicolor flow cytometry (MFC) in an estimate of the proliferative activity of B-cell non-Hodgkin lymphoma by comparing the expression of Ki67 using MFC and immunohistochemicals (IHC).

**Method:**

A total of 559 patients with non-Hodgkin B-cell lymphoma were immunophenotyped using sensitive MFC, of which 517 were newly diagnosed and 42 were transformed lymphomas. Test samples include peripheral blood, bone marrow, various body fluids, and tissues. Through MFC multi-marker accurate gating, abnormal mature B lymphocytes with restricted expression of the light chain were screened. Ki67 was added to determine the proliferation index; the positive rate of Ki67 in tumor B cells was evaluated by cell grouping and internal control. For tissue specimens, MFC and IHC analyses were performed simultaneously to assess the Ki67 proliferation index.

**Results:**

The positive rate of Ki67 by MFC was correlated with the subtype and aggressiveness of B-cell lymphoma. Ki67 could distinguish indolent lymphomas from aggressive subtypes with a cut-off value of 21.25%, and differentiate transformation from indolent lymphoma with a cut-off value of 7.65%. The expression of Ki67 by MFC (regardless of the type of samples)was highly agreement with the Ki67 proliferative index of tissue samples assessed by pathologic immunohistochemistry. MFC showed a fairly constant negative bias in evaluating tissue or bone marrow samples, compared with IHC.

**Conclusions:**

Ki67 is a valuable flow marker that can distinguish between indolent and aggressive types of lymphoma and assess whether indolent lymphomas are transformed. Using MFC to evaluate the positive rate of Ki67 is important in clinical settings. MFC has unique advantages in judging the aggressiveness of lymphoma in samples of bone marrow, peripheral blood, pleural and ascites, and cerebrospinal fluid. This is particularly important when tissue samples cannot be obtained, making it an important supplement for pathologic examination.

## 1 Introduction

Non-Hodgkin B cell lymphoma (B-NHL) is characterized by rearranged immunoglobulin gene and monoclonal B-cell populations and was classified into indolent (approximately 11 subtypes), aggressive (18 subtypes), and very aggressive (2 subtypes) according to the clinical manifestations and outcome (1–3). This grading affects the type of medical treatment; for example, indolent lymphoma could only require a follow-up, while aggressive lymphoma requires immediate medical intervention. Similarly, some chemotherapeutic medicines, such as nitrogen mustard phenylbutyrate and fludarabine, work well for inert lymphomas but have a limited effect on aggressive lymphomas, while enhanced and combined chemotherapy treatments are necessary for very aggressive kinds of lymphoma (4).

MFC is a high-throughput, high-sensitivity tool that can analyze and identify the cell series, degree of differentiation, and abnormal phenotype. It plays an increasingly important role in hematological tumor diagnosis, subtype analysis, and the detection of minimal residual disease (MRD). Subtype diagnosis of B-cell lymphoma is difficult and requires a combination of hemopathology (including tissue and cell morphology, immunophenotype, cytogenetics, and molecular biology) and clinical features to make a precise diagnosis. As a complementary diagnostic method for immunohistochemistry, MFC has the unique advantages of high efficiency, objectivity, and quantitative analysis, and plays an indispensable role in the diagnosis and differential diagnosis of lymphoma, especially for rare primary effusion lymphoma (PEL), chronic lymphocytic leukemia/small lymphocytic lymphoma (CLL/SLL), primary CNS lymphoma (PCNSL), primary vitreoretinal lymphoma (PVRL), autoimmune lymphoproliferative syndrome (ALPS), and complex lymphoma (3, 5, 6). Due to a lack of tissue structure and the fact that different subtypes of mature lymphocytic tumors can exhibit the same or similar immunophenotypes, immunotyping has no advantage over the diagnosis of some subtypes of B-NHLs (e.g., lymphoplasmacytic lymphoma, marginal zone lymphoma, CD5-negative mantle cell lymphoma, CD10-negative follicular lymphoma, and some diffuse large B-cell lymphoma) (5, 6). However, as an important auxiliary tool for pathological diagnosis, the most important task of MFC in detecting mature lymphocytic tumors is to judge the cell characteristics, find tumors that cannot be found using pathological means and judge their aggressiveness. Especially in the initial diagnosis, when bone marrow, peripheral blood, and pleural peritoneal fluid are the first or only samples available for diagnosis, judging the aggressiveness is important when MFC is used to screen abnormal B lymphocytes. However, there is currently no unified standard for judging the aggressiveness of lymphoma by MFC.

The cell proliferation rate is an important factor in grading and predicting the clinical behavior and prognosis of human tumors, especially for B-cell non-Hodgkin lymphoma (B-NHL). There are three main invasive markers of mature B-lymphocyte tumors: Ki67, CD38, and CD71, of which Ki67 has the best sensitivity and specificity (4, 7, 8). Ki67 is a nuclear protein antigen that exists in proliferating cells during the cell cycle (9), and its expression level can be evaluated by the proliferation index (PI=number of cells staining positive for Ki67/total number of cells in the sample) (10). The expression of Ki67 in human tissue is highly correlated with the proliferation rate and can be used to determine the growth fraction of specific cell populations in humans (11).

A pathological immunohistochemical study found that a Ki67 proliferation index greater than 45% is defined as aggressive lymphoma, with a sensitivity of 85% and a specificity of 88.8% (4). MFC can multiparametric characteristic to distinguish pathological cells from normal counterparts, and accurately assess the positive rate of Ki67 in tumor cells through precise gating combined with immunophenotyping. It is suitable for effectively detecting fresh suspension cell samples and can avoid differences in detection caused by different operators. However, there is currently no unified standard data about the sensitivity and specificity of the MFC analysis of Ki67 expression during lymphoma diagnoses. Additionally, alignment between IHC and MFC-derived Ki67 indices has rarely been investigated. This study establishes the expression and investigates the agreement between IHC and MFC when assessing the Ki67 expression/index to evaluate whether MFC could serve as a reliable alternative method for estimating proliferative activity in B-NHL.

## 2 Materials and methods

### 2.1 Study population

Flow cytometric results from patients who were diagnosed with mature B-cell neoplasms from October 2015 to October 2020, were reviewed retrospectively. Each case represented a primary diagnosis of lymphoma that was made based on an incisional or excisional tissue biopsy or fine-needle aspiration biopsy specimens. Histologic slides including immunohistochemical slides, were reviewed without knowledge of the flow cytometric results to confirm the initial diagnoses in all available cases. The diagnosis was made according to the World Health Organization (WHO) 2008 classification (12), WHO 2017 classification,and WHO 2022 classification (2, 3, 13). These patients included 119 patients with DLBCL, 25 patients with Burkitt lymphoma, 67 patients with MCL, 76 patients with follicular lymphoma (FL), 30 patients with marginal zone lymphoma (MZL), 32 patients with lymphoplasmacytic lymphoma (LPL)/Waldenstrom’s macroglobulinemia (WM), 159 patients with chronic lymphocytic leukemia (CLL)/small lymphocytic lymphoma (SLL), 5 patients with hairy cell leukemia, 4 patients with mucosa-associated lymphoid tissue lymphoma (MALT-L), and 42 patients with transformed lymphoma. For the diagnosis, Ki67 expression in lymphoma cells was detected in the bone marrow, pleural effusion, and ascites or lymph node samples. The present study was approved by the Ethical Committee of Tongji Hospital, Tongji Medical College, and Huazhong University of Science and Technology (permit number TJ-IRB20200716), and all procedures conducted followed the protocols of the Declaration of Helsinki.

### 2.2 Flow cytometry—immunophenotyping and Ki67

Mature B-cell lymphoproliferative disease in patients >30yrs was screened by monoclonal antibodies mixed with CD45-V500c, CD19-Pacific Blue, CD20-APC, CD38-PerCP-Cy5.5, CD10-PE-Cy7, kappa-FITC, and lambda-PE. B-cell and plasma cells in patients >30yrs were screened with a tube containing CD45-V500c, CD19-Pacific Blue, CD20-APC-Cy7, CD38-PerCP-Cy5.5, CD56-ECD, CD138-APC, cytoplasmic κ-FITC, and cytoplasmic λ-PE. The samples were all stained with these T- and B-cell lymphatic screening tubes.

This article uses a method of MFC sample staining and panel screening (14). If the screening tube detected that the B lymphocytes had an abnormal immunophenotype, we continued to label fluorescent antibody Ki67; Kappa-FITC/Lambda-PE were purchased from DAKO; CD19-Pacific Blue, and CD20-Pacific Blue were purchased from Biolegend. The BD Pharmingen FITC Mouseanti-Ki67 Set (Clone: B56) is compatible with the BD IntraSure™ Kit (641776), and the remainder was purchased from the Becton Dickinson Company (BD, San Jose, CA). Intracellular staining with Ki67 was performed according to the manufacturer’s instructions. At least 5000 abnormal CDl9+ B lymphocytes or a total of 3×105 white blood cells were obtained from each tube. The abnormal B lymphocyte population was gated by CD45/SSC, CDl9/SSC, CDl9/FSC, CD20/SSC, and CDl9/CD20, and the positive rate of Ki67 in these cells was analyzed. The gating strategy for detecting the positive rate of Ki67 expression in non-Hodgkin B-cell lymphoma by flow cytometry is shown in the **Supplementary Figure**. Abnormal B lymphocytes were defined as populations of CDl9+ or CD20+ cells restricted by Kappa or lambda immunoglobulin light chain expression (or double-negative), abnormal scattered light such as alterations in forwarding scattered light (FSC) and (or) side scattered light (SSC), and abnormal B lymphocytes were often associated with abnormal expression of other antigens, such as CLL cells CD5+CD23+CD20dimCD22dimCD79dim (15). Specimens were analyzed on a BDIS FACSFortessa MFC system from the Becton Dickinson Company and Diva software was used for analysis (BD Biosciences, San Jose, CA).

### 2.3 Ki67 evaluation in histopathology

Immunohistochemistry. Primary antibodies and other auxiliary reagents used for immunohistochemical staining of Ki67 (MIB-1, Dako Cytomation, Glostrup, Denmark) was purchased from Dako. The methods of immunohistochemical staining and assessment of Ki67 PI were those used in this article (4). All analyses were performed by at least two hematopathologists in a single laboratory.

### 2.4 Statistical analysis

Continuous variables were compared using independent group t-tests or one-way ANOVA for normally distributed data. The difference among different types of B-cell lymhoma groups were compared using one-way ANOVA for multiple comparisons. Family-wise error correction with P < 0.05 was significant. *Post-hoc* test with Bonferroni correction was performed to explore the intergroup difference within a mask of ANOVA results. The difference among different types of B-cell lymhoma groups were compared using one-way ANOVA for multiple comparisons. Family-wise error correction with P < 0.05 was significant. *Post-hoc* test with Bonferroni correction was performed to explore the intergroup difference within a mask of ANOVA results. Data were analyzed by the Mann-Whitney U-test or Kruskal-Wallis H-test when the data were not normally distributed. Receiver–operator characteristic (ROC) curves analysis and Youden index of markers were performed to determine the maximum likelihood value that could distinguish transformation lymphoma from indolent lymphoma or aggressive lymphoma. Agreement between Ki67 tested by MFC and immunohistochemistry was assessed by the Passing-Bablock regression analysis. Analyses were performed with SPSS, version 25.0 (IBM Corp., Armonk, NY, USA). The cutoff for statistical significance was P < 0.05.

## 3 Results

### 3.1 Comparison of the positive rate of Ki67 expression in different B-cell lymphomas by MFC

**Figure 1:**
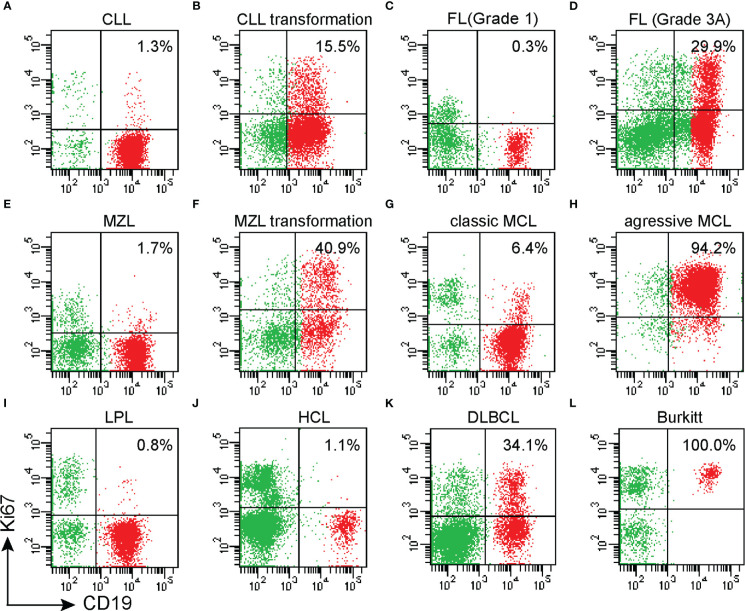
The expression of Ki67 in various non-Hodgkin B-cell lymphomas and transformed B-cell lymphomas was assessed by MFC; **(A)** chronic lymphocytic leukemia (CLL); **(B)** transformation of chronic lymphocytic leukemia (CLL); **(C)** Grade 1 Follicular lymphoma(FL); **(D)** Grade 3A FL; **(E)**. Nodal marginal zone lymphoma (MZL); **(F)** Nodal marginal zone lymphoma (MZL) progressed to Diffuse large B-cell lymphoma (DLBCL); **(G)**. Classic MCL; **(H)** Blastic variant MCL; **(I)** Lymphoplasmacytic lymphoma (LPL); **(J)** Hairy cell leukemia (HCL); **(K)** Diffuse large B-cell lymphoma (DLBCL); **(L)** Burkitt lymphoma.

**Figure 2:**
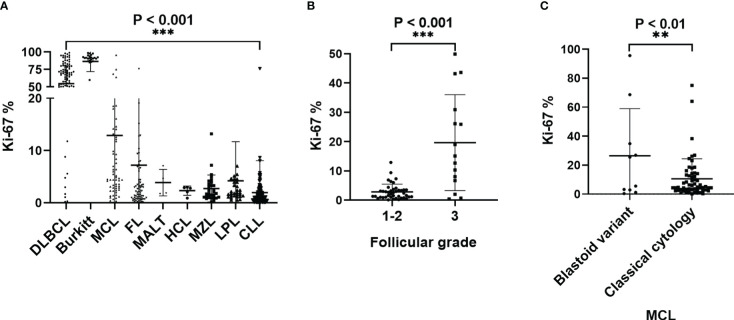
**(A)**The histogram shows the positive rate of Ki67 expression in mature B-cell lymphoma. Burkitt lymphoma Ki67% was higher than all other groups, P<0.001. DLBCL Ki67% was higher than other groups, except for BL cases, P<0.001. BL Ki67% was higher than other groups, except for DLBCL cases, P<0.001 **(B)** The positive rate of Ki67 in FL with pathological grade 3 was significantly higher than in FL with pathological grades 1-2; **(C)** The positive rate of Ki67 expression in aggressive MCL was significantly higher than in classic MCL.Abbreviations: CLL: chronic lymphocytic leukemia; MZL: marginal zone lymphoma; LPL: lymphoplasmacytic lymphoma; MCL: mantle cell lymphomas; FL: follicular lymphoma; HCL: hairy cell leukemia; Burkitt: Burkitt lymphoma; DLBCL: diffuse large B-cell lymphoma. **p < 0.01, ***p < 0.001.

The positive rate of Ki67 in the specimens from 559 patients with B-NHL ranged from 0.1 to 99% (**Table 1**). DLBCL and Burkitt lymphomas had higher Ki67 expression compared with other indolent small cell lymphomas, and Burkitt lymphoma cases had the highest Ki67 percentage. The percentage of malignant cells with DLBCL cases and Burkitt lymphoma cases was 54.8% ± 25.8% and 86.6% ± 14.6%. Very indolent types of lymphoma (MALT lymphoma, Marginal-zone lymphoma, lymphoplasmacytic lymphoma, Chronic lymphoma leukemia/small lymphocytic lymphoma, and Hairy cell leukemia) had the lowest values (3.9, 2.7, 4.2, 2.0, and 2.3%, respectively). In follicular lymphoma, the average positive rate of Ki67 increased with the grade, from 2.0% ± 2.6% in grades 1-2 to 19.6% ± 16.4% in grade 3. Additionally, the positive rate of Ki67 in aggressive mantle cell lymphoma (including pleomorphic and blastic mantle cell lymphoma) was significantly higher than in classical mantle cell lymphoma (26.5% ± 32.5% and 10.5% ± 13.9%, respectively). The positive rate of Ki67 in transformation lymphoma of 42 patients was 35.0% ± 27.5%, which is significantly higher than that of indolent lymphoma (see **Figures 1**, **2**) (**Table 1**).

**Table 1:** The positive rate of Ki67 in patients with Non-Hodgkin Lymphoma (WHO Classification).

Lymphoma type	No. of patients	Ki67 (%) Mean (SD)	Range (%)
DLBCL	119	54.8 (25.8)	0.2-99.0
Burkitt lymphoma	25	86.6 (14.6)	42.1-98.7
MCL	67	12.9 (18.5)	0.1-95.6
Blastoid variant	10	26.5 (32.5)	0.4-95.6
Classical cytology	57	10.5 (13.9)	0.1-75.0
FL	76	7.2 (11.3)	0.1-49.9
Follicular grade 1-2	54	2.0 (2.6)	0.1-12.9
Follicular grade 3	22	19.6 (16.4)	0.4-49.9
MALT	4	3.9 (2.5)	1.8-7.1
MZL	30	2.7 (2.6)	0.2-13.2
LPL	32	4.2 (7.5)	0.5-40.1
CLL/SLL	159	2.0 (6.1)	0.1-8.7
Hairy cell leukemia	5	2.3 (0.9)	0.9-3.0
Transformation	42	35.0 (27.5)	1.0-94.4
CLL/SLL transfer to DLBCL	14		
FL transfer to DLBCL	22		
MZL transfer to DLBCL	2		
Classical cytology of MCL transfer to Blastoid variant	4		

DLBCL, diffuse large B-cell lymphoma; MCL, mantle cell lymphoma; FL, follicular lymphoma; MALT, extranodal marginal-zone lymphoma of mucosa-associated lymphoid tissue; MZL, marginal-zone lymphoma; LPL, lymphoplasmacytic lymphoma; CLL/SLL, chronic lymphoma leukemia/small lymphocytic lymphoma.

### 3.2 Relationship between positive rate of Ki67 expression by MFC and disease grade and disease transformation progression of lymphoma

**Figure 3:**
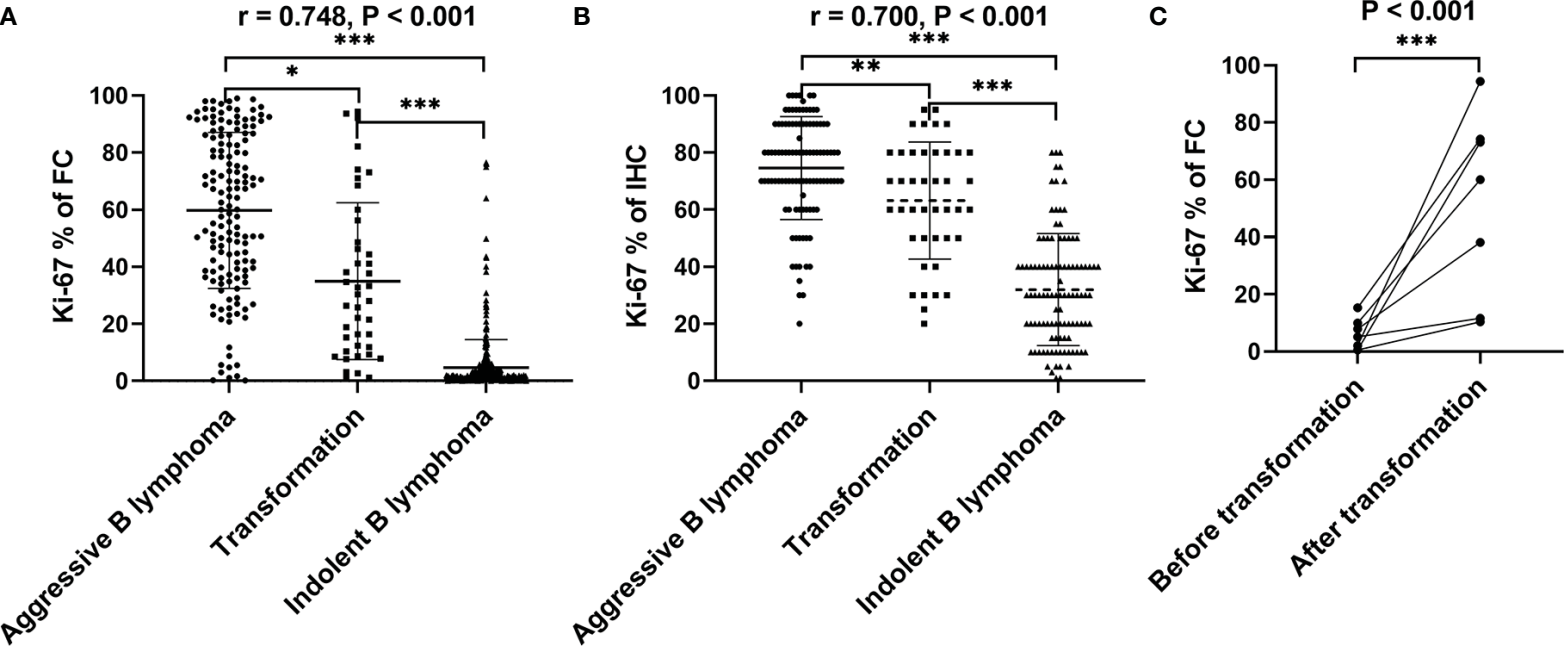
The difference of KI67 expression among aggressive lymphoma, transformed lymphoma and indolent lymphoma. **(A)** The positive rate of Ki67 expression by MFC was significantly different in aggressive B-cell lymphoma, indolent B-cell lymphoma, and transformed B-cell lymphoma. And among the 3 groups of lymphoma, the expression positive rate of Ki67 by MFC was highest in the aggressive lymphoma group, followed by the transformed lymphoma, and the lowest was the indolent lymphoma. **(B)** Pathologically IHC detected Ki67 PI was significantly different in aggressive B-cell lymphoma, indolent B-cell lymphoma, and transformed B-cell lymphoma. Among the 3 groups of lymphoma, the expression positive rate of Ki67 by IHC also was highest in the aggressive lymphoma group, followed by the transformed lymphoma, and the lowest was the indolent lymphoma. **(C)** The positive rate of Ki67 expression in the same patient gradually increased with the progression and transformation of the disease.*p < 0.05, **p < 0.01, ***p < 0.001.

According to the WHO diagnostic criteria, we divided the lymphomas into aggressive B-cell lymphoma, indolent B-cell lymphoma, and transformed 3 groups, and statistically analyzed the difference of KI67 expression positive rate by MFC and IHC among the three groups of lymphoma. MFC showed that the positive rates of Ki67 expression significantly differed between aggressive B-cell lymphoma, indolent B-cell lymphoma, and transformed B-cell lymphoma. And among the 3 groups of lymphoma, the expression positive rate of Ki67 by flow cytometry was highest in the aggressive lymphoma group, followed by the transformed lymphoma, and the lowest was the indolent lymphoma. The positive rate of Ki67 expression was strongly correlated with the aggressiveness of lymphoma, with a correlation coefficient of 0.748 (see **Figure 3A**). Pathological IHC Ki67 PI also showed a significant difference between aggressive B-cell lymphoma, indolent B-cell lymphoma, and transformed B-cell lymphoma. Moreover, among the three groups of lymphomas, the PI of KI67 was also the highest in aggressive lymphomas, followed by transformed lymphomas and the lowest in indolent lymphomas. The positive rate of Ki67 expression was strongly correlated with the aggressiveness of lymphoma, with a correlation coefficient of 0.700 (see **Figure 3B**). In our long-term follow-up visit, we observed that in the seven patients with small indolent B-cell lymphoma undergoing disease progression and transformation, the positive rate of Ki67 expression significantly increased by MFC (see **Figure 3C**).

### 3.3 Receiver operating characteristic curve analysis of the positive rate of Ki67 expression has high sensitivity and specificity in distinguishing indolent and aggressive lymphoma and whether indolent lymphoma has transformed

**Figure 4:**
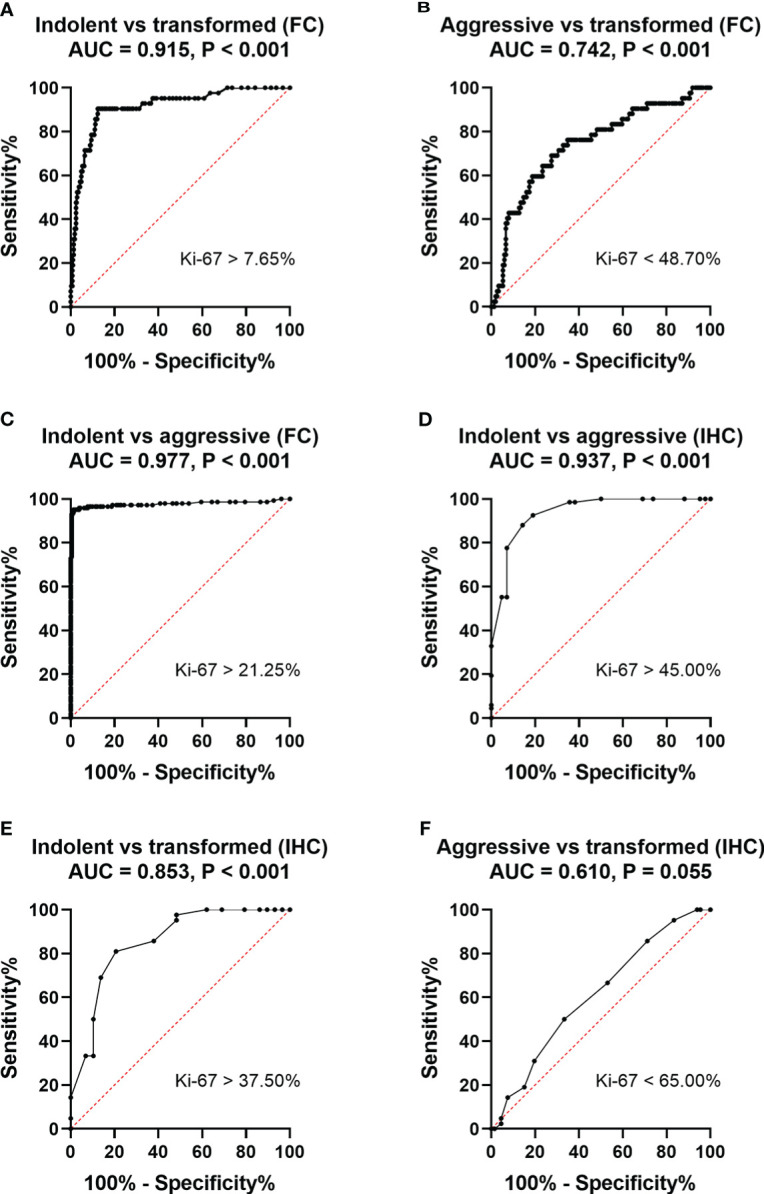
ROC curves for the sensitivity and specificity of the positive rate of Ki67 expression in the diagnosis of non-Hodgkin B-cell lymphoma. A comparison between indolent and aggressive large B lymphomas and transformed B cell lymphoma. **(A)** ROC analysis of the positive rate of Ki67 expression by MFC. Indolent- vs transformed B cell- lymphomas. AUC, 0.915. **(B)** ROC analysis of the positive rate of Ki67 expression by MFC. Aggressive large B cell-vs transformed B cell- lymphomas. AUC, 0.742. **(C)** ROC analysis of the positive rate of Ki67 expression by MFC. Indolent- vs aggressive large B- lymphomas. AUC, 0.977. **(D)**. ROC analysis of the positive rate of Ki67 expression by pathological IHC. Indolent- vs aggressive large B- lymphomas. AUC, 0.937. **(E)**. ROC analysis of the positive rate of Ki67 expression by pathological IHC. Indolent- vs transformed B-cell lymphomas. AUC, 0.853.**(F)** ROC analysis of the positive rate of Ki67 expression by pathological IHC. Aggressive- vs transformed B-cell lymphomas. AUC, 0.610.

The ROC curve analysis established 7.65% as the cut-off value for MFC to distinguishing indolent from transformed B cell lymphoma (area under the curve =0.915, P < 0.001); Therefore, when this value was used, the positive rates of Ki67 expression could identify transformed B cell lymphoma with a sensitivity of 90.5% and a specificity of 87.6% (**Figure 4A**). ROC curve analysis established 48.7% as the cut-off value for MFC to distinguishing aggressive large B cell lymphoma from transformed B cell lymphoma (area under the curve =0.742, P < 0.001); Therefore, when this value was used, the positive rates of Ki67 expression could identify transformed B cell lymphoma with a sensitivity of 76.2% and a specificity of 65.3% (**Figure 4B**). ROC curve analysis established 21.25% as the cut-off value for MFC to distinguishing aggressive from indolent lymphomas (area under the curve =0.977, P < 0.001); Therefore, when this value was used, the positive rates of Ki67 expression could identify aggressive lymphomas with a sensitivity of 93.0% and a specificity of 99.6% (**Figure 4C**). ROC curve analysis established 45.00% as the cut-off value for pathologic IHC to distinguish aggressive from indolent disease (area under the curve =0.937, P < 0.001); Therefore, when this value was used, the positive rates of Ki67 expression could identify aggressive lymphomas with a sensitivity of 92.5% and a specificity of 81.00% (**Figure 4D**). ROC curve analysis established 37.50% as the cut-off value for pathologic IHC to distinguish indolent from transformed disease (area under the curve =0.853, P < 0.001); Therefore, when this value was used, the positive rates of Ki67 expression could identify transformed lymphomas with a sensitivity of 90.9% and a specificity of 64.6% (**Figure 4E**). ROC curve analysis established 65.00% as the cut-off value for pathologic IHC to distinguish aggressive from transformed disease (area under the curve =0.610, P = 0.055); Therefore, when this value was used, the positive rates of Ki67 expression could identify transformed lymphomas with a sensitivity of 50.0% and a specificity of 66.7% (**Figure 4F**).

### 3.4 Agreement analysis of the positive rate of Ki67 expression by MFC and Ki67 proliferation index by pathological immunohistochemistry

**Figure 5:**
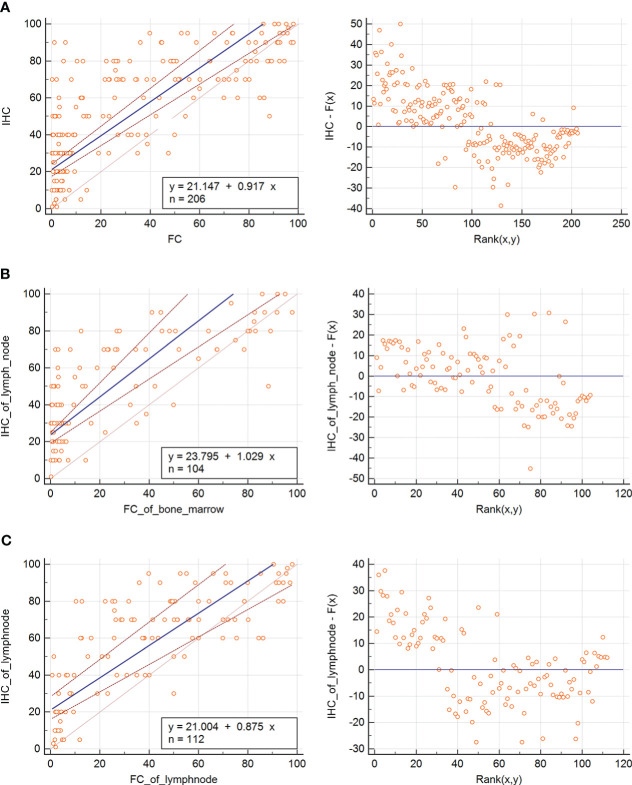
Passing-Bablock regression analysis of MFC Ki67 expression and IHC Ki67 index. **(A)** The Passing–Bablock linear regression analysis showed a good agreement between the two methods regardless of sample type. The formula of regression equations was: IHC = 21.147 (95% CI: 17.482 - 24.042) + 0.917 (95% CI: 0.832 - 1.027) × FC (P<0.01). **(B)** The Passing–Bablock linear regression analysis showed also a good agreement between Ki67 from bone marrow sample detected by FC and Ki67 from lymph node sample detected by IHC. The formula of regression equations was: IHC of lymph node = 23.795 (95% CI: 19.260 - 25.075)+ 1.029 (95% CI: 0.868 - 1.345) × FC of bone marrow (P<0.01). **(C)** The Passing–Bablock linear regression analysis showed also a good agreement between Ki67 from lymph node detected by FC and Ki67 from lymph node detected by IHC. The formula of regression equations was: IHC = 21.004 (95% CI: 16.060 - 28.327) + 0.875 (95% CI: 0.743 - 1.010) × FC (P=0.01).

MFC results in positive Ki67 expression and showed significant differences in the positive Ki67 rates between highly aggressive and indolent lymphoma (P < 0.001), transformed B-cell lymphoma, and indolent lymphoma (P < 0.001). Given that pathology is the gold standard for diagnosing lymphoma, we used Passing-Bablock regression analysis as a statistical method to analyze the agreement between the positive expression of Ki67 detected by MFC and the proliferation index of Ki67 detected by pathological immunohistochemistry.The Passing–Bablock linear regression analysis showed a good agreement between the two methods regardless of sample type (**Figure 5A**). The formula of regression equations was: IHC = 21.147 (95% CI: 17.482 - 24.042) + 0.917 (95% CI: 0.832 - 1.027) × FC (P<0.01). Additionally, we statistically analyzed sample types separately, and the results showed also a good agreement with pathologic immunohistochemistry regardless of the flow cytometric detection of bone marrow samples or tissue samples. The Passing–Bablock linear regression analysis showed also a good agreement between Ki67 from bone marrow sample detected by FC and Ki67 from lymph node sample detected by IHC(**Figure 5B**). The formula of regression equations was: IHC of lymph node = 23.795 (95% CI: 19.260 - 25.075)+ 1.029 (95% CI: 0.868 - 1.345) × FC of bone marrow (P<0.01). The Passing–Bablock linear regression analysis showed also a good agreement between Ki67 from lymph node detected by FC and Ki67 from lymph node detected by IHC (**Figure 5C**). The formula of regression equations was: IHC = 21.004 (95% CI: 16.060 - 28.327) + 0.875 (95% CI: 0.743 - 1.010) × FC (P=0.01).

## 5 Discussion

Over the past few decades, Ki67 has been of great significance in evaluating the proliferation index (Ki67 index) in lymphoma, and its importance and correlation in the diagnosis, prognosis, and prediction of treatment response have been confirmed (4, 16, 17). Chan JK et al. reported in a retrospective study that the Ki67 positive index was closely related to the Revised European-American Classification of Lymphoid Neoplasms (REAL), and its proliferation index was associated with poor clinical prognosis (18). The Southwest Oncology Group in the United States considered Ki67 to be an essential index for judging the prognosis of NHL as early as the 1990s (19). Nowadays, immunohistochemistry of Ki67 MIB-1 positive in paraffin tissue sections has proved to be the most commonly used method to measure the growth fraction of benign and malignant cell populations. Patients with non-Hodgkin lymphoma that have a high expression of Ki67 have a greater likelihood of malignant behavior, poor survival, and poor prognosis (2, 4, 18).

The relationship between the Ki67 proliferation index and the clinical grade and type of lymphoma has been previously studied using immunohistochemistry. Landberg and Roos evaluated Ki67 expression in non-Hodgkin lymphoma (NHL) cells and the percentage of expression allowed for the classification of high and low-grade lymphomas (20). Data from PP Brons et al. showed that Ki67 was strongly correlated with histological classification in the three malignant lymphoma grades: a low percentage of neoplastic proliferating cells in low-grade lymphomas and significantly higher numbers in intermediate- and high-grade lymphomas (21). Adi Broyde et al. assessed the diagnostic (subclassification and grading) and prognostic value of Ki67 PI for malignant lymphoma. When examining the distribution of Ki67 proliferation index expression in tissue samples from 319 newly diagnosed lymphoma patients (WHO subcategory), they noted that the mean Ki67 PI differs by type of lymphoma (4). Therefore, most studies found consistent results in the relationship between Ki67 expression and the clinical grade and type of lymphoma; the higher the grade of lymphoma, the higher the Ki67 expression (22). MFC has been increasingly used in recent years in the nuclear antigen diagnosis of lymphoma from blood and bone marrow aspirates and lymph node fine-needle aspirates (6, 23), but the significance of using MFC to detect Ki67 expression has not been well elucidated. Our study focused on comparing the use of MFC to detect the Ki67 index in B-NHL with pathological immunohistochemistry and evaluated the reliability of MFC in estimating the aggressiveness of B cell lymphoma. Our results showed that the two methods were highly correlated in all samples and that the positive rate of Ki67 expression by MFC was strongly correlated with the aggressiveness of lymphoma.

The Ki67 proliferation index is strongly correlated with the grade and subtype of lymphoma. In the WHO classification of lymphoma, the Ki-67 positive index is associated with the pathological grading and prognosis of follicular lymphoma and is an auxiliary marker for the pathological grading of follicular lymphoma (2, 24, 25). In mantle cell lymphoma, the degree of proliferation of tumor cells is considered to be a strong predictor of biological prognosis (26–28). Guidelines published by the European Mantle Cell Lymphoma Pathology have standardized the assessment of the Ki67 index by immunohistochemical methods for routine applications (29). Cases with < 10% Ki67-positive cells have a more indolent course. Blastoid variants with increased Ki67 expression are associated with a more aggressive clinical course (30, 31). Data from our study is consistent with previously reported histopathology trends. In our study, the positive rate of Ki67 differed between different (follicular lymphoma) grades, and the Ki67 positive rate increased with the grade of lymphoma. The positive rate of Ki67 in aggressive mantle cell lymphoma (including pleomorphic and blast variant mantle cell lymphoma) was significantly higher than in classical mantle cell lymphoma. This data demonstrates that the positive expression rate of Ki67 detected by MFC plays an important role in the staging and subtype diagnosis of lymphoma, and can help identify the subtype of the disease.

Since some types of B-NHL can exhibit different clinical behaviors, it is necessary to identify rapid clinical progression as early as possible and adjust treatment accordingly (32, 33). It is particularly important to identify patients with indolent lymphoma but is high risk for transformation lymphoma because aggressive therapy can alter the treatment outcome (34). Previous studies have demonstrated that the Ki67 index is one of the most useful indicators for the early recognition of disease progressionand for detecting the transformation of indolent lymphoma to aggressive lymphoma before it is evident in clinical manifestation (34). In our research, we analyzed the positive rate of Ki67 in 42 patients with indolent lymphoma transformation. The results demonstrated that there was a significant difference in the positive rate of Ki67 detected by MFC among aggressive B-cell lymphoma, indolent B-cell lymphoma, and transformed B-cell lymphoma. Additionally, seven patients were initially diagnosed with indolent small B-cell lymphoma that later progressed during treatment follow-up. The positive expression rate of Ki67 gradually increased with the transformation of indolent small B lymphoma to aggressive large B lymphoma (**Figure 3C**). The above results showed that multi-color flow cytometry could simultaneously analyze the cellular immune phenotype and the positive rate of Ki67, which played an important role in the diagnosis and identification of lymphoma progression.

The Ki67 index, as detected by IHC, was routinely used to identify the aggressiveness of B-NHL and the cut-off value has been addressed. Many studies have assessed the cut-off value of the Ki67 positive rate. Hall PA et al. retrospectively found that there was a strong correlation between a low Ki67 index (< 20%) and low-grade histology according to the Kiel classification of NHL and a high Ki67 index (> 20%) and high-grade histology (16). Landberg G et al. (35) found that the majority of low-grade lymphoma cases had <5% Ki67-positive cells and most high-grade lymphomas had >20%. Additionally, in previous studies on histologic sections, Fournel-Fleury et al. reported a similar cut-off (21%), although this was a study on canine lymphoma (36). In our study, the cut-off value for distinguishing indolent lymphoma from aggressive lymphoma was 21.25%, and 7.65% for distinguishing indolent lymphoma from transformed B-cell lymphoma by MFC. The data from our study are similar to those of previous reports. Analysis of the positive rate of Ki67 in tumor cells using these two values, in combination with immunophenotyping specificity, revealed a distinction between indolent and aggressive lymphoma and could tell whether or not indolent lymphoma was transformed, meaning it has good sensitivity and specificity. The sensitivity and specificity of identifying transformed B-cell lymphoma and aggressive large B-cell lymphoma with Ki67 are slightly lower because there is a large overlap in the positive rate of Ki67 between the two diseases, which must be further confirmed along with other relevant examinations, such as pathology, molecules, and FISH.

Except for the previously mentioned cut-off values for immunohistochemical analyses, which were similar to our study of MFC, there are studies reporting a higher cut-off value data for the Ki67 index for immunohistochemical analyses. Broyde et al. evaluated the histochemical Ki67 index in 319 patients with newly diagnosed NHL. The mean Ki67 index significantly increased from 26.6% in indolent lymphomas to 67.2% in aggressive lymphomas to 97.6% in very aggressive lymphomas. They established a Ki67 index of 45% to differentiate indolent from aggressive lymphomas (4). Some other investigators have also analyzed the usefulness of the Ki67 index in tissue aspirate specimens using immunohistochemistry. In a study involving 86 tissue aspirate specimens of non-Hodgkin lymphoma, a cut-off value of 38% was established to distinguish indolent from aggressive lymphoma (37). The cut-off values of these studies all exceeded 21.25%. The cut-off value of the pathologic IHC Ki67 proliferation index in our hospital for distinguishing indolent from aggressive lymphomas was 45%, consistent with that used by Broyde et al (4). We established with ROC curve analysis 37.50% as the cut-off value for differentiating indolent from transformed lymphoma and 65.00% as the cut-off value for distinguishing aggressive from transformed disease by pathologic IHC, and it could be seen that these cut-off values for IHC were all higher than those for MFC. Our results showed that the Ki67 positive rate by MFC analysis in the same patient was in good agreement with the proliferation index of tissue samples assessed by pathological immunohistochemistry. However, while the two methods were consistent, MFC showed a fairly constant negative bias across the observation range when evaluating either tissue samples or bone marrow samples.

There are several possible explanations for this negative bias. First, Ki67 is a nuclear antigen, and we used the conventional BD IntraSure™ Kit as a permeabilizing agent for flow staining, which could penetrate the nuclear membrane to improve the positive rate. Second, in this study, different Ki67 clones in MFC and IHC (B56 in MFC and MIB-1 in IHC, respectively) could be another reason for the inconsistent results. The comparative analysis results of the different clones showed that the clonal indices of MIB-1 and B56 in IHC were significantly higher than those of the other four tested clones, with no significant difference between the B56 antibody and MIB-1 (38, 39). In immunohistochemistry, WHO guidelines recommend the assessment of Ki67 positive cells in the most proliferative areas of the slide, which are selected by the evaluators. This could result in observational bias. Additionally, microscopic differentiation between specific and non-specific staining could be difficult in IHC, while in MFC a cell suspension reflecting the average proliferative activity is assessed. Further, MFC allows simultaneous immunophenotyping of proliferating living cells, circumventing non-specific antibody binding as such aiding the discrimination between neoplastic and nonneoplastic cells. The positive rate of Ki67 by multicolor flow cytometry was delineated according to the isotype control and internal control, and the results were more objective. Finally, eight cases of DLBCL in the bone marrow specimens were examined, belonging to a type of discordant lymphoma (40, 41). Discordant lymphoma occurs where 2 distinct histologic subtypes coexist in at least 2 separate anatomic sites. Histologic discordance is most commonly observed between the bone marrow (BM) and lymph nodes (LNs), while aggressive lymphoma is typically found in an LN biopsy with indolent lymphoma in a BM biopsy. This phenomenon could also explain why MFC has a smaller overall negative bias value for tissue samples than for bone marrow.

## 6 Conclusions

In conclusion, Ki67 is a valuable flow marker that can distinguish between indolent and aggressive lymphomas and identify whether indolent lymphomas are transformed, and using MFC to evaluate the positive rate of Ki67 is important in clinical settings. The positive rate of Ki67 was correlated with the subtype and malignancy of lymphoma and was also highly positively correlated with the pathological immunohistochemical proliferation index. Since the Ki67 staining analysis by MFC is fast, objective, reliable, inexpensive, and can be combined with immunophenotypes, it has unique advantages in judging the aggressiveness of lymphoma in samples of bone marrow, peripheral blood, pleural and ascites, and cerebrospinal fluid, particularly when tissue samples cannot be obtained. Therefore, it is an important supplement for pathologic examinations.

## Data availability statement

The raw data supporting the conclusions of this article will be made available by the authors, without undue reservation.

## Ethics statement

The studies involving human participants were reviewed and approved by Ethics Committee, Tongji Hospital, Tongji Medical College, Huazhong University of Science and Technology (permit number TJ-IRB20200716), Wuhan, China. The patients/participants provided their written informed consent to participate in this study.

## Author contributions

XM designed, wrote and submitted this manuscript. YL collected clinical data and made statistical analysis to make statistical charts. YL and CW revised the manuscript. SL, CH, SY and LZ were responsible for data collection and flow cytometry. DK is mainly responsible for pathological diagnosis and immunohistochemical staining. MX mainly discussed the manuscript. All authors contributed to the article and approved the submitted version.

## Supplementary material

Supplementary figure of B-NHL gating This is a lymph node specimen from a patient with progressive transformation of marginal zone B-cell lymphoma detected by flow cytometry. The living cells P1 and lymphocytes were gated first, and then B lymphocytes were gated by SSC/CD19. B lymphocytes were divided into two groups in CD19/CD20 diagram. The blue cells were polyclonal normal B lymphocytes, and the red cells were monoclonal abnormal B lymphocytes and did not express kappa and lambda. According to the homotype control and cell grouping, the positive rate of Ki67 expression in abnormal B lymphocytes was 38.5%.
