# Supplementary figures and images for: Multicolor flow cytometric assessment of Ki67 expression and its diagnostic value in mature B-cell neoplasms
Source: Front Oncol. 2023 Feb 17;13:1108837. doi: 10.3389/fonc.2023.1108837 (PMC9986934; doi:10.3389/fonc.2023.1108837)

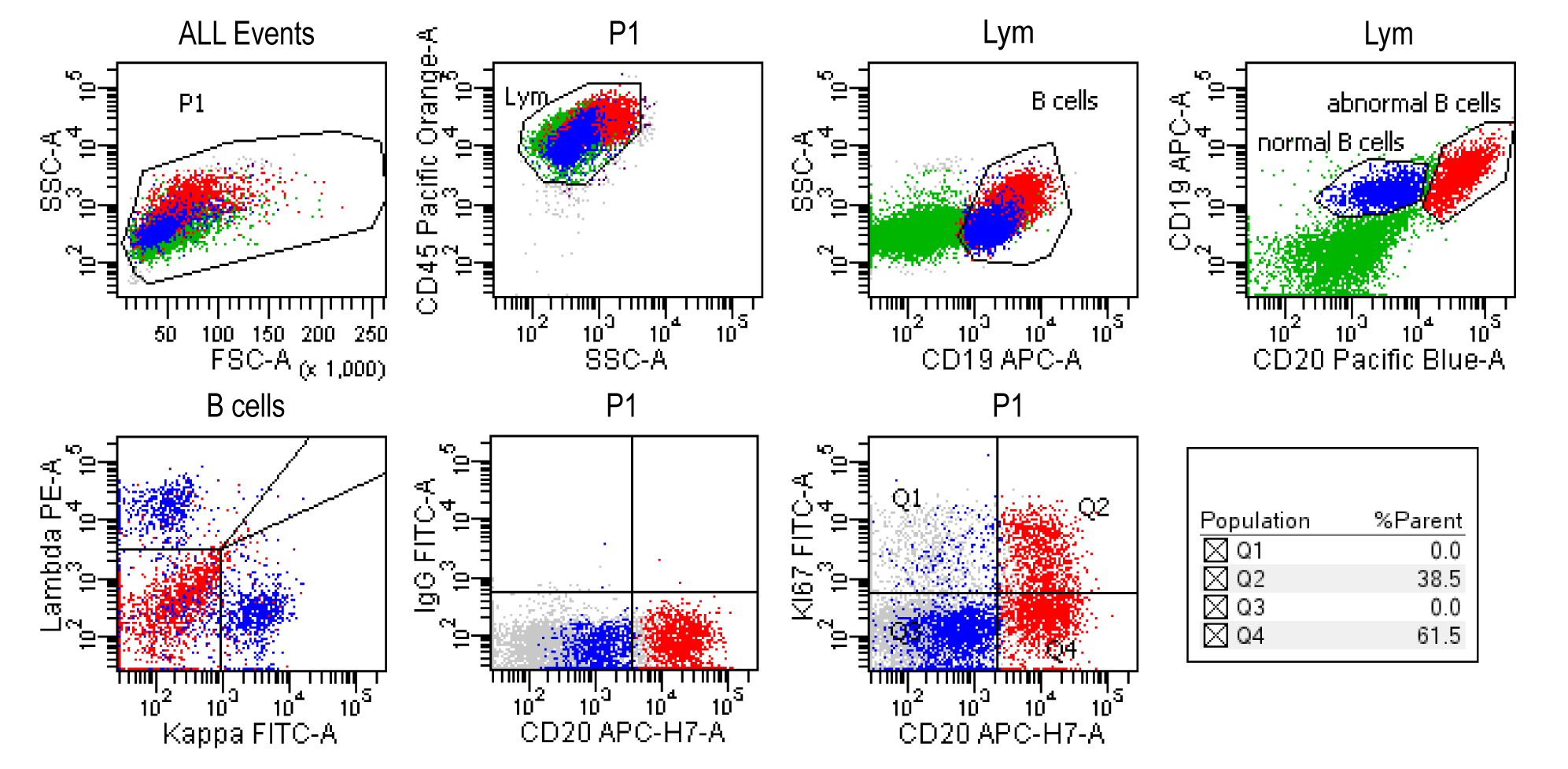

Supplement: Supplementary figure of B-NHL gating — This is a lymph node specimen from a patient with progressive transformation of marginal zone B-cell lymphoma detected by flow cytometry. The living cells P1 and lymphocytes were gated first, and then B lymphocytes were gated by SSC/CD19. B lymphocytes were divided into two groups in CD19/CD20 diagram. The blue cells were polyclonal normal B lymphocytes, and the red cells were monoclonal abnormal B lymphocytes and did not express kappa and lambda. According to the homotype control and cell grouping, the positive rate of Ki67 expression in abnormal B lymphocytes was 38.5%. [file Image_1.tif]
